# Evaluation of wound healing effect of alginate film containing *Aloe vera* gel and cross-linked with zinc chloride ^1^

**DOI:** 10.1590/s0102-865020200050000007

**Published:** 2020-07-03

**Authors:** Adriana Yuriko Koga, Julio César Felix, Rodrigo Gomes Marques Silvestre, Leandro Cavalcante Lipinski, Bruna Carletto, Fernanda Alexia Kawahara, Airton Vicente Pereira

**Affiliations:** I Fellow PhD degree, Postgraduate Program in Pharmaceutical Sciences, Universidade Estadual de Ponta Grossa (UEPG), Brazil. Technical procedures, analysis and interpretation of data, manuscript writing.; II Professor, Director in Technology Institute of Paraná, Curitiba-PR, Brazil. Scientific, conception and design of the study.; III Master, Industrial Director in Technology Institute of Paraná, Curitiba-PR, Brazil. Scientific, conception and design of the study.; IV PhD, Associate Professor, Department of Medicine, UEPG, Ponta Grossa-PR, Brazil. Design of the study, technical procedures, statistical analysis, interpretation of data, critical revision, final approval.; V Fellow PhD degree, Postgraduate Program in Pharmaceutical Sciences, UEPG, Ponta Grossa-PR, Brazil. Technical procedures, manuscript writing.; VI Master, Postgraduate Program in Biomedical Science, UEPG, Ponta Grossa- PR, Brazil. Design of the study, technical procedures.; VII PhD, Associate Professor, Department of Pharmaceutical Science, UEPG, Ponta Grossa-PR, Brazil. Design of the study, technical procedures, interpretation of data, manuscript writing, critical revision, final approval.

**Keywords:** Wound Healing, Aloe, Zinc, Rats

## Abstract

**Purpose:**

To develop a new wound dressing composed of alginate and Aloe vera gel and cross-linked with zinc ions.

**Methods:**

The aloe-alginate film was characterized using scanning electron microscopy (SEM), swelling profile, mechanical properties, polysaccharide content and X-ray diffraction (XRD). Thirty Wistar rats were divided in two groups a) treated with aloe-alginate film and b) control (treated with sterile gauze). Wound contraction measurements and hystological analysis were performed on 7th, 14th and 21st days after wound surgery.

**Results:**

The aloe-alginate film presented adequated mechanical resistance and malleability for application as wound dressing. There was no statistical difference in wound contraction between two groups. Histological assay demonstrated that aloe-alginate film presented anti-inflammatory activity, stimulated angiogenesis on proliferative phase and a more significant increased in collagen type I fibers and decreased type III fibers which promoted a mature scar formation when compared to control.

**Conclusions:**

The aloe-alginate film showed adequate physicochemical characteristics for wound dressing applications. The *in vivo* assay demonstrated that aloe-alginate film enhanced the healing process of incisional skin wounds.

## Introduction

Chronic wounds such as diabetic ulcers, pressure ulcers and venous ulcers affect the quality of life of patients and have a great economic and social impact. The cost increases with the population aging and the incidence of chronic diseases like obesity and diabetes^1^ .

The wound healing process involves sucessive phases such as inflammation (platelets activation, recruitment of leukocytes and release of inflammatory mediators), proliferation (keratinocytes and fibroblasts) and maturation or remodeling^2^ .

The high cost and long time of treatment of chronic wounds lead to development of new dressing materials. The requirements of an ideal wound dressing include accelerate healing, high exudate absorption, easily handled and removed, bacterial barrier and costless^3^ .

Alginate is an anionic biopolymer extracted from brown algae. Chemically is a linear copolymer of L-guluronic acid and D-manuronic acid linked in different proportions and sequences. It has been extensively used as wound dressing material because of its mucoadhesive and biocompatibility behavior and non-immunogenic properties^4^ .

Alginate can form different types of structures such as films, particles, hydrogel and extended release microspheres^5^ . The most important characteristic of alginate is its ability to form a hydrogel, a cross-linked structure that absorbs and retains wound exudate and has hemostatic properties^6^ .

Hydrogels of alginate can be obtained by using different techniques. The most common is the ion exchange between Ca^2^ ions and the G blocks of the polymer^5^ . Alginate films can be cross-linked with several cations, such as Ca^2^, Mn^2^, Zn^2^ and Al^3^. Cross-linking extensively affects the physicochemical and mechanical properties of alginate films^7^ .

Zinc is a chemical micronutrient essential for human health with great biological relevance^8^ . Its abundant in the epidermis and the topical zinc sulphate has been used in wound care to enhance healing.

The effect of zinc ion has been demonstrated in wound healing^9^ . The evidence of its role in wound repair is supported by the demonstration of zinc metalloenzymes such as alkaline phosphatase, RNA and DNA polymerases and matrix metalloproteinases (MMPs)^10^ .

Zinc can modulate innate and adaptative immune functions, using mechanisms ranging from lymphocyte differentiation and production of antibodies such as inflammatory signaling^11^ .

Aloe vera ( *Aloe barbadensis* Miller) is a perennial plant that has been reported to have important pharmacological activities, such as anti-inflammatory, analgesic, antioxidant, antineoplastic and wound healing^12^ .

Aloe gel is rich in phenolic compounds and polysaccharides. Acemannan, the major polysaccharide of *Aloe vera* gel, is an acetylated glucomannan that stimulate macrophages proliferation and tissue reepithelization^13^ .

In this study we presented the development and evaluation of the physicochemical properties and the wound healing effect of an alginate film containing Aloe gel and cross-linked with Zn^2^.

## Methods

### Plant identification and development of aloe-gel film

The *Aloe vera* leaves were collected from the garden of Universidade Estadual de Ponta Grossa. The plant was identified as *Aloe barbadensis* Miller and a voucher specimen was deposited in the herbarium under number 22131. The gel was extracted from fresh leaves and kept frozen at -4^o^C. Immediately before the use aloe gel was thawing at room temperature. The alginate film was obtained as described by Koga *et al* .^14^ . A sodium alginate solution was prepared by dissolving the powder (1.6%) in deionized water and then glycerol (plasticizer) (6%) and the *Aloe vera* gel (40%) were added. The mixture was homogenized, transferred to a petri dish and dried at 40 °C for 24 h. The resulting alginate film was cross-linked with ZnCl_2_ solution and left to dry at 40°C for 12h.

### Content of polysaccharide in Aloe gel aloe-alginate film

The content of polysaccharide in the aloe gel samples was determined immediately after collection and after freeze-thawing cycle. The quantification was performed by phenol-sulfuric spectrophotometric method with absorbance readings at 490 nm. Samples of the aloe-alginate film (4 cm^2^) were held for 30 min in 20 mL of distilled water at 37°C until the complete swelling. An aliquot of 500 μL was transferred into glass tube and then 0.5 mL of phenol solution 5% (w/v) and 2.5 mL of concentrated sulfuric acid were added. The resulting solution was kept in an ice bath for 30 minutes and the absorbance was measured at 490 nm.

### Film characterization

#### Mechanical properties

The mechanical properties were evaluated using rectangular strips (13.6 x 25 mm) of cross-linked and non-cross-linked aloe-alginate films in a Shimadzu® R AG-I testing machine with a crosshead speed of 5mm min^-1^.

#### Swelling profile

Cross-linked aloe-alginate film samples (30 mg) were kept in a stainless steel basket dipped in 20 mL of distilled water at 37°C. The film sample was weighed before the test and then again at 10, 20, 30, 60, and 120 min after the removal of excess water. The percentage of swelling was calculated by the weight difference between the hydrated and dried samples.

## Scanning electron microscopy (SEM-FEG)

SEM analysis was performed using a MIRA3 LM, Tescan Orsay Holdingn electron microscope. Prior to analysis, the film samples (cross-linked and non-cross-linked films) were covered with a thin layer of gold by spraying and disposing of a maximum distance to avoid damage to the surface. All the samples were examined using an accelerating voltage of 10 kV.

## X-ray diffraction (XRD)

X-ray diffraction of cross-linked and non-cross-linked aloe-alginate films was performed using an Ultima IV/Rigaku. A scanning rate of 5°/min and range of 3-90° were applied at room temperature.

## Wound healing assay

All the experiments were approved by the Ethics Committee on Animal Experimentation of UEPG, registry number 041/2017. Thirty male Wistar rats (250 g) were divided into the following two groups (n=15): (a) aloe-alginate film and (b) control (sterile gauze). The rats were anesthetized with an i.p. injection of ketamine (40 mg/kg) and xylazine (5 mg/kg).

The backs of the animals were shaved and, under aseptic conditions, a cutaneous wound of approximately 4 cm^2^ was performed at the dorsum using a scalpel. The wounds of all the animals were covered with elastic bandage and that were removed after seven days. The bandage was removed on 3rd day; however, the aloe-alginate films remained adhered to the wounds until the day of euthanasia (the films were no changed). The animals were housed invidually and received water and food “ad libitum”.

Five of each group were euthanized on the 7th, 14th, and 21st post-operative days with an overdose of anesthetic (ketamine and xylazine). Tissue samples were collected and fixed in 10% buffered formalin for subsequente histological analysis.

### Wound contraction

The areas (mm^2^) of the wounds of each animal were registered using a camera (Canyon EOS) with standardized parameters as support, animal position, distance, pixels and ligth. The measurements were taken every seven days and then calculated using Image J software. The wound contraction was calculated using the following equation:

$$\%\;\mathrm{Wound}\;\mathrm{contraction}\;=\;\frac{A1\;–\;A2}{A1}\;x\;100$$

Where, A1 and A2 are the initial wound area and wound area after the pos-operative days, respectively.

## Histological analysis

Tissue of wounds were collected on 7th, 14th, and 21st days and included in paraffin. The tissues samples were cut at a thickness of 3 μm and stained with hematoxylin-eosin. Inflammatory changes and angiogenesis were then observed in three fields under 40 times magnification using a conventional light microscope Olympus. The images were captured using cellSens Standard software. Cell counter image J software was used to count inflammatory cells and blood vessels.

### Picrosirius red

The collagen content was analyzed using picrosirius stained samples on the 14th and 21st days. To observe the collagen fibers the sections were viewed under crossed polarization conditions using a polarization microscope (Olympus LG-PS2). The collagen fibers were analyzed according to their birefringence pattern. The optimal threshold for the positive pixels that corresponded to the areas of type I collagen (red) and type III collagen (green) was determined using Image J software. A binary image was produced and the proportional collagen area was expressed in pixels.

## Statistical analysis

The statistical analysis was performed using analysis of variance (ANOVA) for multiple comparisons, followed by Tukey’s test with a confidence interval of 95% (p ≤0.05).

## Results

### Film characterization

The total polysaccharide determination was obtained using phenol-sulfuric method. The results showed a little decrease of polysaccharide content after freeze-thawing cycle compared with fresh aloe gel. The aloe-alginate film cross-linked with zinc released 54.6% of the polysaccharides contained in the formulation within 30 min.

**Figure 1:**
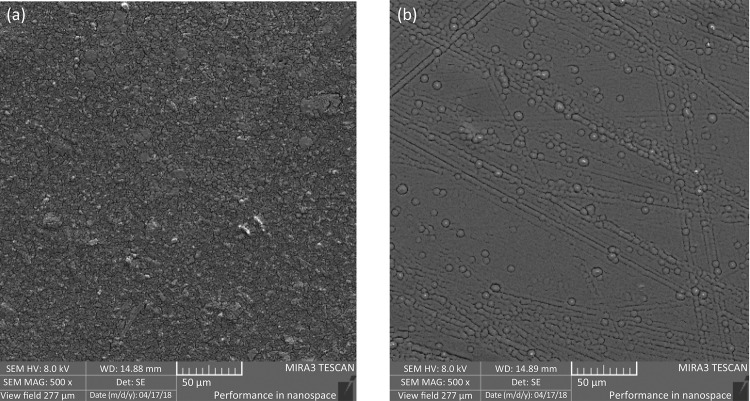
Surface images obtained using SEM micrographs of cross-linked (a) and non-cross-linked (b) aloe-alginate films.

The surface images of the cross-linked aloe-alginate films ( Fig. 1a ) and non-cross-linked ( Fig. 1b ) were obtained by SEM. The micrographs revealed differences between aloe-alginate films. The cross-linked film present higher rough surface when compared with non-cross-linked that presented a smoother surface.

**Figure 2:**
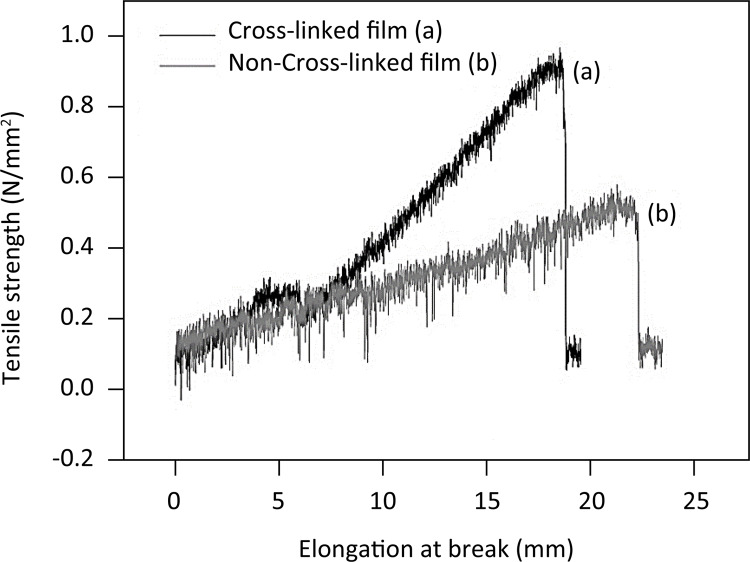
Tensile strength and elongation break of cross-linked (a) and non-cross-linked aloe-alginate (b) films.

Figure 2 illustrates the results for tensile strength and maximum elongation for aloe-alginate films. The aloe-alginate film cross-linked with zinc chloride showed higher strength required for rupture and lower elongation when compared with non-cross-linked film.

**Figure 3:**
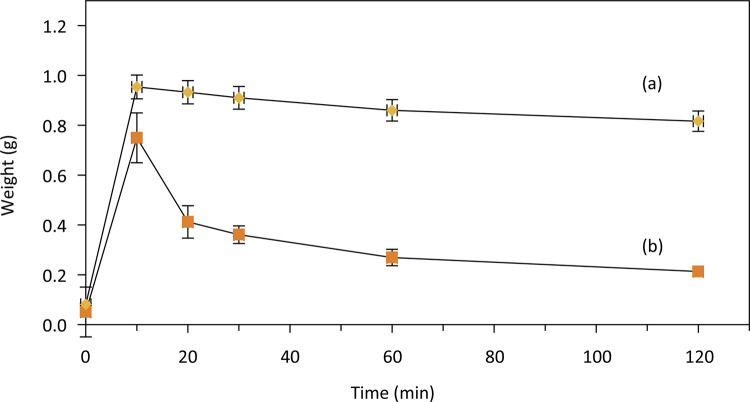
Swelling profiles of cross-linked (a) and non-cross-linked (b) aloe-alginate films.

The swelling assay was performed with non-cross-linked and cross-linked aloe-alginate films. The higher swelling degree and a considerably reduced solubility in water were observed with aloe-alginate film cross-linked witn Zn^2^ ( Fig. 3 ).

**Figure 4:**
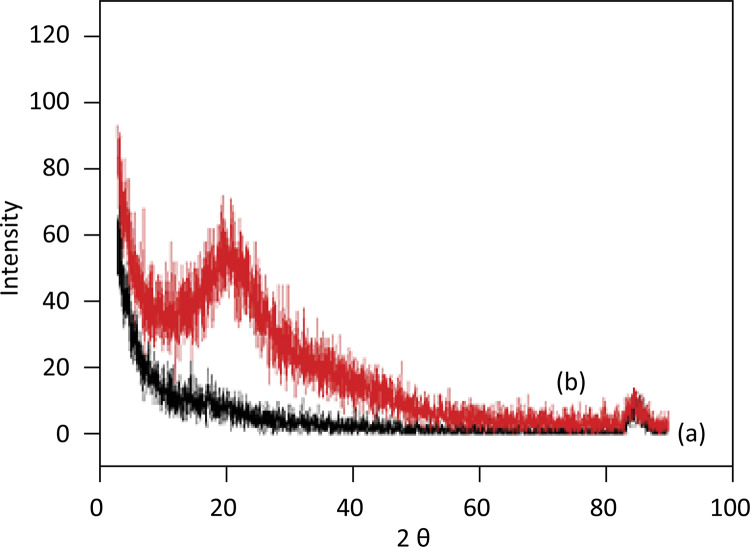
X-ray diffractograms of the aloe-alginate films: cross-linked with ZnCl2 (a) and non-cross- linked (b).

XRD pattern was observed for both non-cross-linked and cross-linked aloe-alginate films ( Fig. 4 ). X-ray data showed that both aloe-alginate films have amorphous characteristics but the small difference (peak at 20.4) in the diffraction pattern of the aloe-alginate film cross-linked with Zn^2^ could be attributed to modification in the arrangement of alginate structure in “egg-box”.

### Wound healing

**Figure 5:**
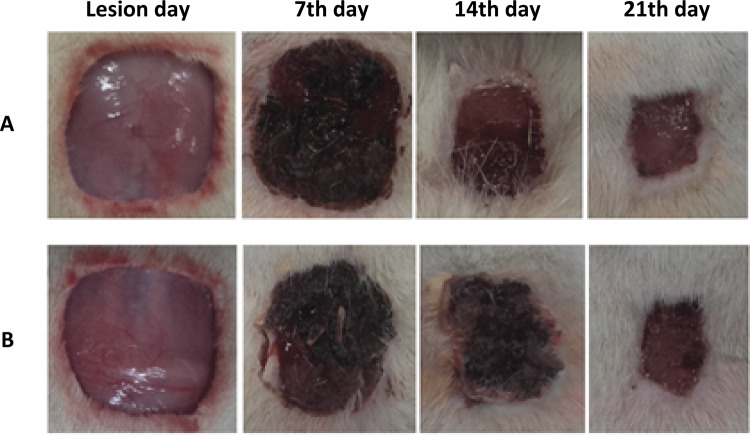
Scared area of skin treated with aloe-alginate film (group A) and sterile gauze (group B) after wounding (0) and days 7, 14 and 21 pos-surgery.

In aloe-alginate group and control the wound contraction was studied by comparing wound areas on 7th, 14th and 21st days ( Fig. 5 ). The percentage of wound contraction was 80.6 and 79.5% to aloe-alginate film and control, respectively.

**Figure 6:**
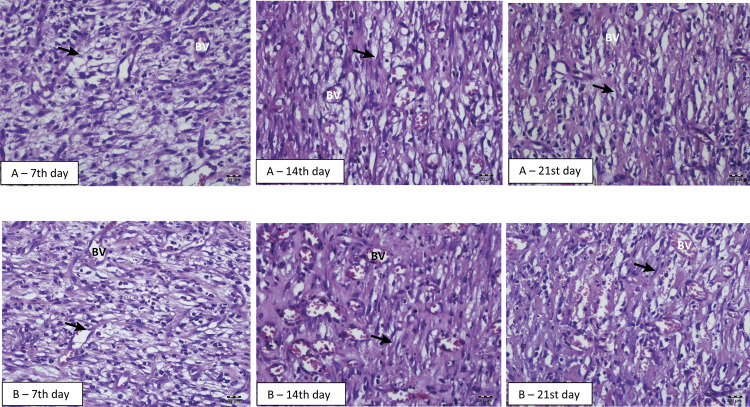
Photomicrograph of the tissue wounds stained with hematoxylin-eosin treated with aloe-alginate film (A) and control (B) on 7th, 14th, and 21st days. The presence of inflammatory infiltrate was observed in all groups (à) and new blood vessels were presented on 7th, 14th and 21st days (BV).

Histological analysis showed that there was an acute inflammatory response on 7th day and the presence of angiogenesis on 14^th^ and 21st days for both groups ( Fig. 6 ).

**Figure 7:**
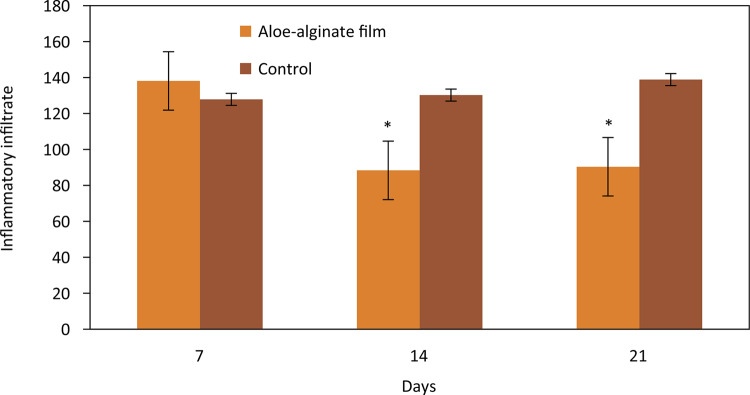
Inflammatory infiltrate in wounds treated with aloe-alginate film and control on 7th, 14th and 21st days. *p≤0.05.

The quantitative analysis of the inflammatory infiltrate presented a significative decrease of inflammatory infiltrate on 14th and 21st days on aloe-alginate group ( Fig. 7 ).

**Figure 8:**
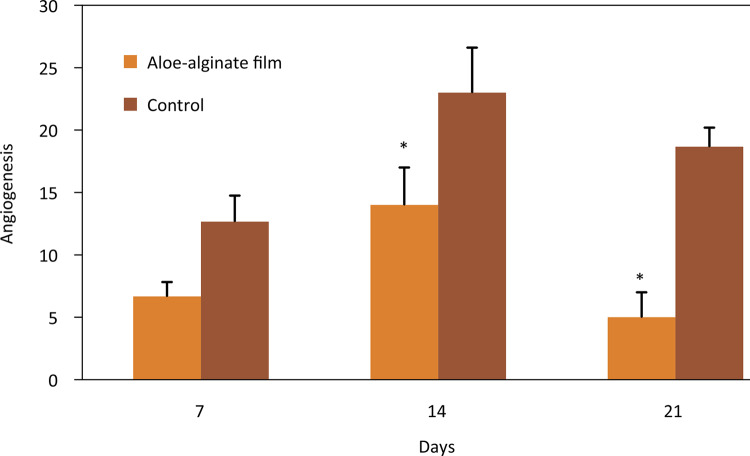
Presence of blood vessels on groups treated with aloe-alginate film and control on 7th, 14th and 21st days. *p≤0.05.

Quantitative analysis of angiogenesis showed that during the wound healing the total number of new blood vessels was lower in the group treated with aloe-alginate film. Although an increase occurred in both groups on 14th day, a significant decrease was observed on 21st day in the group treated with aloe-alginate film ( Fig. 8 ).

**Figure 9:**
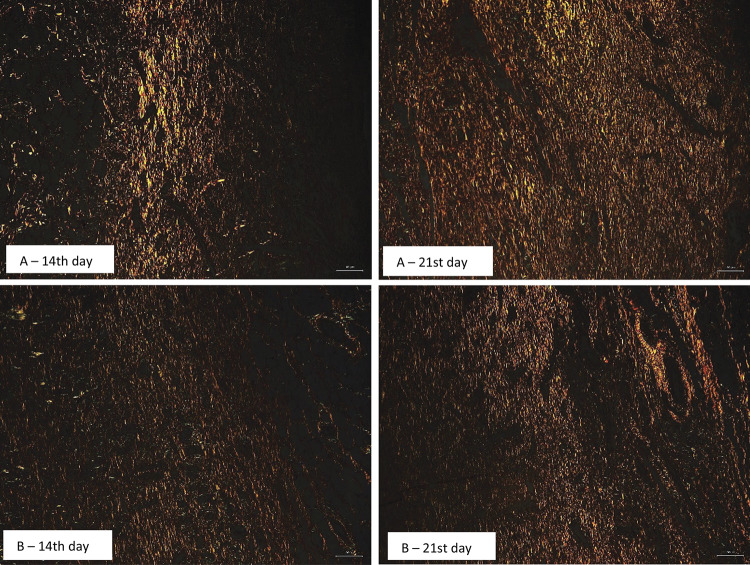
Photomicrograph of type I and type III collagen fiber stained with picrosirius red on groups treated with aloe-alginate film (A) on 14th day and 21st day and control (B) on 14th and 21st day. The differentiation of the fibers was done using a binary image on Image J software.

The total type I and type III collagen were measured by the picrosirius red staining. The collagen type I presents a brillant red color and type III a green color when stained with picrosirius. The disorganized type III collagen is present on the 14th day and uniform type I collagen on 21st day in both groups ( Fig. 9 ).

**Figure 10:**
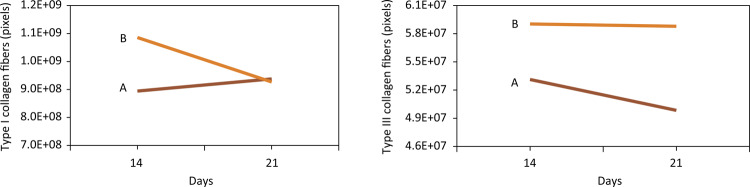
Quantity in pixels of collagen type I and III in the groups treated with aloe-alginate film (A) and control (B) on 14th and 21st days.

The quantitative analysis of collagen fibers showed a more significant increase in collagen type I and a decrease in collagen type III in the group treated with aloe-alginate film on 21st day compared to control group ( Fig. 10 ).

## Discussion

*Aloe vera* ( *Aloe barbadensis* Miller) is a popular medicinal plant for wound healing. Aloe gel contains about 98% of water and polysaccharides constitute 55% of dry matter^15^ . Acemannan, the major polysaccharide extracted from *Aloe vera* gel, can stimulate macrophages activation and promote the release of cytokines from fibroblasts^16^ . The beneficial properties of acemannan in wound healing have been reported^17^ .

Freezing decelerates chemical and physical degradation and is widely used to maintain stability and quality of bioproducts. The polysaccharide content did not differ significantly in both *fresh* and after freeze-thawing aloe gel. The ratio of polysaccharide released from the aloe-alginate film was also determined. The hydrated aloe-alginate film cross-linked with Zn^2^ released 54.6% of polysaccharide from the aloe gel incorporated in the formulation in 30 min. The result of releasing test of polysaccharide evidenced that aloe-alginate film as wound dressing can release these active compounds in the wound bed.

The non-cross-linked and cross-linked aloe-alginate films were visually homogeneous, but SEM micrographs show an increase in roughness of the film cross-linked with zinc. The changes in surface roughness can be attributed to the formation of bonds between Zn^2^ and alginate chains.

The flexibility and elasticity of polymeric films are particularly useful for wound dressings. Alginate films for this purpose needs to be flexible, easily handled and allows pain-free dressing changes. The mechanical properties are influenced by the crosslinking rate and a high ionic concentration lead to an increase of tensile strength and a decrease of elongation at brake^18^ . Aloe-alginate film was obtained by solution casting technique and further crosslinked with zinc chloride. As might be expected, the crosslinking process with Zn^2^ solutions resulted in a decrease in elongation at break (28%) but an increase in tensile strength (90%) compared with non-cross-linked aloe-alginate film. The cross-linked film presented adequate elasticity for application as wound dressing.

The swelling assay showed that cross-linked film had a higher capacity (1090% in 10 min) to absorb water and a lower solubility when compared with the non-cross-linked film. Costa *et al* .^18^ proposed that the higher swelling index of cross-linked alginate films can be explained by the number of alginate strands linked with ions to permit the formation of the “egg-box” conformation. An appropriate ionic concentration is enough to decrease water solubility and maintain a number of strands available to uptake water leading to high values of swelling index.

The result of swelling assay is interesting for wound dressing applications due to the capacity of the cross-linked aloe-alginate film to absorb great amount of exudate and maintain a moist environment for wound healing. Alginate hydrogels are an excellent choice for wound dressing because they are non-adherent, control the dehydration and stimulate cell migration and proliferation^19^ .

The XDR spectra of cross-linked and non-cross-linked aloe-alginate films exhibited amorphous characteristics. The non-cross-linked aloe-alginate film showed a typical peak at 2 teta = 10-30°^20^ . Amorphous characteristics of hydrogel films can provide good adhesion on wound bed and maintain a moist environment for healing^21^ .

### Animal assay

After a single application, no difference in the wound contraction between treatments was observed. Despite this, aloe-alginate film has several advantages over conventional dressings such as higher capacity of exudate absorption, transparency permits continuous observation of the wound bed, acts as a ‘second skin’ protecting the wound and can adapt to wounds located in awkward sites.

The previous work demonstrated that aloe-alginate film cross-linked with calcium had a superior effect on modulation of inflammatory phase, increasing angiogenesis and collagenesis when compared with alginate film without aloe gel^14^ .

Myofibroblasts is an intermediated cell between myocytes and fibroblasts that can produce matrix components and generate contractile forces favoring wound closure^22^ . The group treated with aloe-alginate film presented a decrease of inflammatory infiltrated on 14th (p=0.05) and 21st (p=0.028) days when compared to control group. This result can be attributed to the compounds presented in aloe gel polysaccharides and amino acids that can promote cells mitosis thereby accelerating healing and stimulating macrophages to excrete dead tissue^23^ .

Active compounds of aloe gel can contribute to the anti-inflammatory response. Aqueous extract of *Aloe vera* demonstrated anti-inflammatory effect at the dose of 500mg/kg in acute, sub-acute and chronic models of inflammation^24^ .

*Aloe vera* with chitosan nanoparticle thin-film membranes accelerated healing of incisional infected wounds in rats^24^ . In another recent study, healing effect of aloe gel was evaluated in dorsal wounds of diabetic rats and improved the re-epithelialization rate^25^ .

The zinc ions used in cross-linked process can contribute to modulate inflammatory infiltrate and the proliferation phase. Zinc ion is related to various phases of wound healing such as cofactor to many metalloenzymes, immune defense, re-epithelialization of fibrosis and scar formation^11^ . Wound healing of hydrogel cross-linked with zinc ion was studied by Zhou *et al* .^26^ . The *in vivo* assay demonstrated that treatment with hydrogel promoted fibroblast migration, antibacterial activity, faster wound closure and higher vascularization.

New blood vessels are formed during the proliferative phase to offer sufficient oxygen and nutrients amounts to the wound tissue and reestablish the blood flow^27^ . Quantitative analysis of angiogenesis showed that aloe-alginate film promote an increase in the number of blood vessels on 14th day (p=0.005) following by a decrease on 21st (p=0.002) day compared to control. This result can be associated with the presence of glycoproteins that stimulate proliferative activity of microvessels^28^ . Zinc and calcium ions can stimulate migration of endothelial cell to injury during the wound healing^26^ .

The histological analysis of picrosirius red showed that there was a significant decrease of type III collagen and an increase of type I collagen on 21st day in the group treated with aloe-alginate film compared to control. This results can be attributed to the activity of aloe gel and zinc ion in promote fibroblast migration. Synthesis of collagen in skin burns was stimulated by aloe gel that significantly promoted the formation of a mature scar^29^ . Zinc ion can contribute to migration of fibroblast cells. Alginate polyacrylamide hydrogel cross-linked with zinc ion improved collagen deposition, mature granulation tissue and better re-epithelization of the wound bed^26^ .

The low-dose radiation combined with topical application of fibroblast growth factor and zinc accelerated wound healing of skin lesions in diabetic rats resulting in more organized collagen fibers, faster wound closure and epithelialization^30^ .

## Conclusions

Aloe-alginate film presented adequate physicochemical characteristics for application as wound dressing. The new aloe-alginate film cross-linked with zinc chloride modulated inflammatory phase, increased angiogenesis on proliferative phase and collagen type I suggesting that aloe gel combined with zinc ions contributed to the wound healing process and a better scar formation.
